# Written discourse in diagnosis for acquired neurogenic communication disorders: current evidence and future directions

**DOI:** 10.3389/fnhum.2023.1264582

**Published:** 2024-01-11

**Authors:** Hana Kim, Jessica Obermeyer, Robert W. Wiley

**Affiliations:** ^1^Department of Communication Sciences and Disorders, University of South Florida, Tampa, FL, United States; ^2^Department of Neurology, The Johns Hopkins University School of Medicine, Baltimore, MD, United States; ^3^Department of Communication Sciences and Disorders, University of North Carolina at Greensboro, Greensboro, NC, United States; ^4^Department of Psychology, University of North Carolina at Greensboro, Greensboro, NC, United States

**Keywords:** written discourse, aphasia, primary progressive aphasia, mild cognitive impairment, writing assessment, Alzheimer’s disease

## Abstract

**Purpose:**

We aimed to perform the first review of research focusing on written discourse performance in people with acquired neurogenic communication disorders. In studies from 2000 onward, we specifically sought to determine: (1) the differences between patient populations and control groups, (2) the differences between different patient populations, (3) longitudinal differences between patient populations, and (4) modality differences between spoken and written discourse performance.

**Methods:**

We completed a thorough search on MEDLINE, Embase, Cochrane, APAPsycinfo, Web of Science, and Scopus databases. We identified studies that focus on written discourse performance in people with aphasia, primary progressive aphasia, mild cognitive impairment, and Alzheimer’s disease.

**Results:**

Nineteen studies were identified from the review of literature, some of which addressed more than one of our review questions. Fifteen studies included a comparison between clinical populations and controls. Six studies compared different characteristics of patient populations. Three studies reported changes over time in progressive disorders. Six studies targeted different modalities of discourse.

**Conclusion:**

Differences in linguistic features by patient populations are not yet clear due to the limited number of studies and different measures and tasks used across the studies. Nevertheless, there is substantial evidence of numerous linguistic features in acquired neurogenic communication disorders that depart from those of healthy controls. Compared to the controls, people with aphasia tend to produce fewer words, and syntactically simpler utterances compared to the controls. People with Alzheimer’s disease produce less information content, and this feature increases over time, as reported in longitudinal studies. Our review imparts additional information that written and spoken discourse provide unique insights into the cognitive and linguistic deficits experienced by people with aphasia, Alzheimer’s disease, mild cognitive impairment and primary progressive aphasia and provide targets for treatment to improve written communication in these groups.

## 1 Introduction

Writing plays an essential role in everyday communication. Traditionally, people use writing in a variety of situations, such as writing letters and making lists (Ball and Postman, 2022). Much of the writing that is required for daily communication happens at the discourse level (e.g., emails, electronic messaging, notes), which is defined as language production via speaking or writing (handwriting or typing) beyond the phrase level (Ulatowska and Olness, 2004). Advances in technology mean that writing has become the primary medium of communication through mobile technologies and the internet. However, writing is inherently complicated with the integration of multiple cognitive and linguistic aspects required to deliver a message (Graham et al., 2007). Due to the complexity of processing required for writing, it has been suggested that brain injuries and neurological disease often result in deficits that impact writing (Wilson and Proctor, 2002; Rapp and Fischer-Baum, 2015). Although writing is becoming more and more essential for the completion of activities of daily communication, it is underrepresented in the acquired language disorders literature especially at the discourse level. This review paper provides a summary of the existing research on written discourse in people with aphasia (PWA), primary progressive aphasia (PPA), Alzheimer’s disease (AD), and Mild Cognitive Impairment (MCI). Given the increasing importance of writing in daily life, this review provides a starting point which can inform future research and clinical practice.

Within the frame of cognitive psychology, Hayes and Flower presented a theoretical model to describe writing processes in which three recurrent phases were assumed to take place: planning, generating, and revision (Hayes and Flower, 1980). The planning phase implies writers’ ability to set goals for how to organize the knowledge in response to the topic of the writing activity, while the generating phase usually denotes the actual writing. During the revision phase, writers revisit their writing, and make changes in the text. This model highlights that all of the phases are orchestrated to accomplish written discourse with cognitive systems such as working memory. As this model suggests, written discourse represents a complex level of language production. In a recent qualitative study, eight individuals with aphasia were interviewed about their writing experience (Thiel and Conroy, 2022). Although they were generally frustrated by things like the slow progress and limited content they could produce, they felt happy when they were able to write in real-word situations (e.g., greeting cards and emails). This reflects that for writing, real-world communication is meaningful, and more functional than word-level communication.

One of the specific goals of this review paper is related to the diagnostic sensitivity of written discourse. This goal was addressed by reviewing the literature to identify documented differences in written discourse between PWA, PPA, AD, and MCI and people without acquired language disorders, as well as characteristics of discourse-level writing performance that distinguish different types of acquired language disorders. A second goal was to determine if written discourse analysis is sensitive to measuring change across time, which is especially relevant for future research on writing intervention, and to measure changes in written language in progressive acquired language disorders like AD, MCI, and PPA. We also evaluated the literature on differences between spoken and written discourse, which is relevant to how written discourse is analyzed for the purpose of clinical and research practice. The completion of these goals provides information that is relevant to clinical and research practice in terms of identifying targets for treatment in each population, assisting with differential diagnosis of neurodegenerative communication disorders, and providing methods to evaluate response to treatment or changes in language function over time. Additionally, we provide a summary of elicitation tasks and measures that are currently being used to assess and evaluate written discourse performance, which will provide preliminary information about the state of written discourse research.

### 1.1 Written discourse as a diagnostic tool

There is a substantive body of literature on the methods of spoken discourse analysis and characteristics of spoken discourse in acquired neurogenic communication disorders (Bryant et al., 2016; Pritchard et al., 2017; Mueller et al., 2018; Suárez-González et al., 2021). There is also a subset of that literature that seeks to evaluate the diagnostic sensitivity of spoken discourse measures as it relates to identifying specific populations in comparison to groups without acquired language disorders (Fleming and Harris, 2008; Kim et al., 2019) and from different clinical populations (Glosser and Deser, 1992; Ash et al., 2013; Fromm et al., 2017). One of the aims of this study is to review the existing literature to determine which written discourse measures consistently distinguish our populations of interest from performance of adults without acquired language disorders and thus may be diagnostically sensitive in a clinical environment. We also summarized the literature to determine how written discourse characteristics differed when compared across populations of people with acquired language disorders. Doing this provides insight into which measures may be relevant when evaluating the written discourse of specific clinical populations and provides important groundwork for written discourse research.

### 1.2 Using discourse to measure change over time

Performance on discourse tasks reflects the status of progression or recovery in neurological disorders (Brisebois et al., 2021; Kim et al., 2022a,b). Using discourse to monitor longitudinal changes is clinically compelling in that discourse is a sample that is easily accessible for repeated investigations (Ahmed et al., 2013). Discourse samples provide rich information with cost-effective, non-invasive methods (Horigome et al., 2022). Unlike the prevalence of research into spoken discourse ability, there has been less focus on the utility of written discourse to identify clinically meaningful changes. Thus, this review summarized extant evidence that documents how well written discourse is able to capture changes in language and cognitive function that may occur over time in populations with progressive acquired language disorders (AD, MCI, PPA). This topic is also relevant to measuring change after treatment.

### 1.3 Written vs. spoken discourse

In the written domain, discourse can encompass email, messaging platforms, letters, etc. Historically, spoken discourse has received much more attention than written discourse because most daily communication took place in the spoken modality. In the spoken modality this can include telling stories, giving directions, and participating in conversation. However, the increased use of technology to complete activities of daily living means that people, including people with acquired language disorders, encounter more situations that require writing in the form of typing. For example, common activities such as shopping and banking may now be completed online, and many social outlets can be accessed virtually (social groups, messaging, etc.). These changes mean that people with acquired language disorders are more likely to need discourse level writing skills, especially in the form of typing. However, there is limited research on how to evaluate and treat written language at the discourse level.

Currently, when written discourse is assessed, it is done with tasks and measures that were originally created to evaluate spoken language, due to the lack of specific tools for evaluating written discourse in people with acquired language disorders (Obermeyer and Edmonds, 2018; Jaecks and Jonas, 2022). Further, there is limited research on how to interpret performance and potential changes in written discourse. This lack of information specific to written discourse is problematic because of the known differences between written and spoken discourse performance. For example, it is known that individuals *without* acquired language impairments tend to use more complicated language in writing than in speaking, and the complexity of writing increases up to adolescence (Wilson and Proctor, 2002). Spoken discourse is more spontaneous and does not allow an opportunity to edit and try out (Behrns et al., 2009), which differentiates it from written discourse. Further, spoken and written languages are developmentally and evolutionarily viewed as independent, and the two language systems are not in a subservient relationship (Pulgram, 1965; Vachek, 1989). Due to the heterogeneous nature of the two modalities and differences in their neural substrates, spoken and written discourse may be differentially affected in people with acquired neurogenic communication disorders and thus writing needs to be specifically evaluated and treated in the clinical environment.

### 1.4 The need to focus on writing

As we have stated, functional communication increasingly requires written discourse, which means there is an increasing need to focus of writing assessment and treatment earlier in the rehabilitation of people with acquired language disorders. Behrns et al. (2010) also point out that discourse level writing is often not targeted in aphasia assessment or treatment, potentially due to how difficult it is for PWA to produce written discourse, and that many people with aphasia are discharged from speech-language services by the time they would be ready to address written discourse. Given the increased need to write/type in daily life, a paradigm shift is required in research and clinical work to meet the needs of people with acquired language disorders and make it possible for them to access multiple modalities of communication. This shift also corresponds to the International Classification of Functioning, Disability, and Health (ICF) framework that clinical assessments should encompass individuals’ ability to participate in daily activities (World Health Organization, 2007). Thus, this study examined the state of the current literature on written discourse in adults with acquired language disorders, in order to provide evidence of language specific features that can be potentially used for diagnosis and target treatment. Because of how limited the research literature in this area is, we sought to answer specific questions that can be used to inform future research.

### 1.5 Aims

The goal of this study was to review the extant literature to determine the current status of knowledge related to written discourse performance in adults with select, acquired neurogenic communication disorders (people with aphasia, primary progressive aphasia, mild cognitive impairment, and Alzheimer’s disease). This review aims to summarize the evidence of written discourse deficits in clinical populations, focusing on clinically distinct linguistic features that characterize language impairments. Additionally, we extend our review to examine modality differences between spoken and written discourse and research that has used written discourse to evaluate change across time. Specifically, we sought to determine if current research supports the use of written discourse as a sensitive diagnostic measure that should be incorporated into clinical assessment. In order to capture the current state of written discourse research we also included a summary of the measures and elicitation tasks that can guide clinical practice by identifying tasks and measures that are commonly used to evaluate written discourse. In support of this goal, we identified four research questions:

(1) What are the documented differences in written discourse when comparing PWA, PPA, MCI, and AD to control groups?
(2) Does performance on written discourse tasks/measures distinguish between different patient populations?
(3) Are there written discourse measures that can be used to evaluate change over time in progressive neurogenic communication disorders?
(4) How does written discourse performance compare to spoken discourse performance in people with acquired neurogenic disorders?

## 2 Methods

### 2.1 Systematic search

Prior to narrative review, a systematic search was conducted in order to improve the methodological rigor (Grant and Booth, 2009; Ferrari, 2015). The literature search was completed on MEDLINE, Embase, Cochrane, APAPsycinfo, Web of Science, and Scopus databases. The search was run on February 26, 2023. The literature search was completed by research librarians at Johns Hopkins University, following guidelines described by Cochrane. Two main concepts were combined in the search: types of acquired neurogenic communication disorders, and written discourse. The search summary using keywords for the concepts can be found in Supplementary Appendix 1.

### 2.2 Screening and data extraction

Covidence Systematic Review Software (covidence.org) was used for the screening process. Two reviewers (HK, JO) independently screened titles and abstracts based on inclusion/exclusion criteria. Full texts of articles were obtained for studies that met the inclusion criteria or where abstracts were not sufficient to determine eligibility. The full text articles were then reviewed independently against selection criteria by two reviewers. The conflicts from both abstract and full-text screenings were resolved by a third reviewer (RW).

Articles met inclusion criteria only if they (1) focused on written discourse performance, (2) studied linguistic aspects of written discourse, (3) used discourse elicitation tasks that are replicable in clinical settings, (4) tested the target patient populations (people with aphasia, primary progressive aphasia, mild cognitive impairment, or Alzheimer’s disease), and (5) were peer-reviewed articles published since the year 2000. Studies with participants with mental health issues or other neurological conditions (e.g., Parkinson’s disease) were not included. Studies evaluating only motoric skills of writing were also excluded.

**FIGURE 1:**
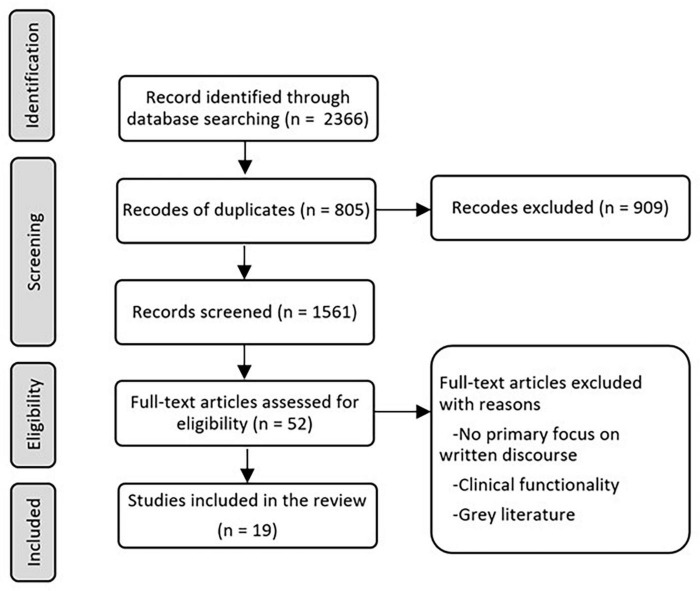
Path diagram describing identification, screening, eligibility, and inclusion.

Following the screening process and application of inclusion criteria, we extracted relevant information related to target population, discourse tasks used, linguistic measures used, and findings. Our primary focus of this review was to gather data that reveal potential diagnostic sensitivity of written discourse in individuals with acquired neurogenic communication disorders, identify written discourse measures that are appropriate for capturing the characteristics unique to each clinical population, and to provide methods to evaluate change over time. Results of the search process are presented in Figure 1.

## 3 Results

### 3.1 Study characteristics

A total of 2,366 articles were identified through the literature search. Following the removal of duplicates, 1,561 articles were reviewed at the abstract screening stage. Later, 52 articles were used for full text screening, and a total of 19 studies were identified for inclusion in the review.

Of the studies we reviewed, 15 articles evaluated differences between clinical populations and controls. Six of the papers reviewed addressed written discourse in PWA as compared to a control group (Mortensen, 2005; Behrns et al., 2008, 2009, 2010; Vandenborre et al., 2018; Johansson-Malmeling et al., 2020). Seven articles sought to compare the written discourse of people with Alzheimer’s disease to that of a control group using a variety of discourse elicitation tasks and measures (Forbes et al., 2004; Forbes-McKay and Venneri, 2005; Pekkala et al., 2013; Forbes-McKay et al., 2014; Rodríguez-Ferreiro et al., 2014; Davy et al., 2016). Two additional papers compared the written discourse of people with MCI to that produced by control groups (Aramaki et al., 2016; Smolík et al., 2016), and one paper compared multiple levels of writing produced by people with MCI, AD, and a control group (Hayashi et al., 2015). Identified deficits compared to controls can be targets for treatment. Unfortunately, there were no studies investigating linguistic characteristics in the written discourse performance of people with PPA compared to controls.

Six articles compared differential characteristics of patient populations cross-sectionally. Hayashi et al. (2015) focused on the comparison between people with amnestic MCI and AD, while Forbes et al. (2004), Groves-Wright et al. (2004), and Sand Aronsson et al. (2020) examined the characteristics of written discourse at different stages of AD (subjective cognitive impairment, probable, minimal, mild, moderate AD). Mortensen (2005) examined writing skills of people with aphasia when writing an informal letter to someone whom the study participants were close with. Three other studies conducted a longitudinal investigation on changes in written discourse performance. Sitek et al. (2015) expanded the scope of the patient population by adding people with logopenic PPA. These preliminary data might be helpful in differential diagnosis.

Three studies were specifically interested in changes over time in progressive neurogenic disorders (Pekkala et al., 2013; Forbes-McKay et al., 2014; Kim et al., 2022b). These studies provide assessments to measure change, which might also be applicable for measuring response to treatment.

Six articles targeted the relationship between the written and spoken modalities of discourse: two studies of PWA (Behrns et al., 2009; Vandenborre et al., 2018), two focused on AD (Groves-Wright et al., 2004; Forbes-McKay and Venneri, 2005), and another two studies examined MCI (Aramaki et al., 2016; Smolík et al., 2016).

### 3.2 Participant characteristics

In the 19 articles reviewed, a total of 1321 participants were included. These participants were comprised of 671 healthy controls, 84 people with aphasia, 9 people with logopenic primary progressive aphasia, 28 people with subjective cognitive impairment, 177 people with mild cognitive impairment (amnestic mild cognitive impairment = 94; multiple domain mild cognitive impairment = 5; unidentified type of mild cognitive impairment = 78), 290 people with Alzheimer’s disease, 47 unidentified individuals (either mild cognitive impairment or Alzheimer’s disease), and 15 people with traumatic brain injury. Three articles by Behrns et al. (2008, 2009, 2010) appear to include the same 8 PWA based on the demographics disclosed.

Nine articles out of 19 included participants whose native language is not English. Five articles included native Swedish speakers for both healthy controls, subjective cognitive impairment, MCI, AD, and PWA; and 2 articles included native Japanese speakers for the control group and the MCI group. Native Dutch speaking PWA and the control groups, and native Spanish probable AD group and controls were included in 2 articles, respectively.

Eleven articles matched age and years of education between participants with communication disorders and their counterparts. Three articles in total did not match age and education between groups (Forbes-McKay and Venneri, 2005; Aramaki et al., 2016; Sand Aronsson et al., 2020). Five articles did not mention whether age or education was matched among the groups (Forbes et al., 2004; Behrns et al., 2008, 2009, 2010; Davy et al., 2016).

### 3.3 Written discourse tasks

**FIGURE 2:**
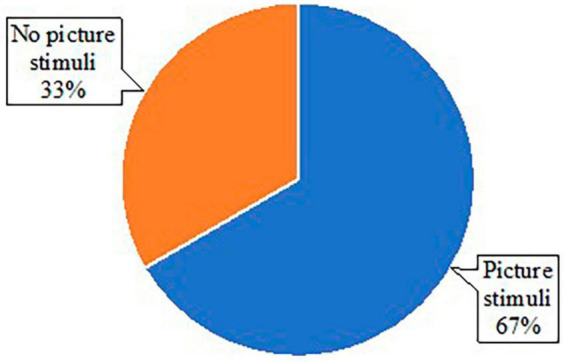
Pie chart of types of stimuli used across studies.

**FIGURE 3:**
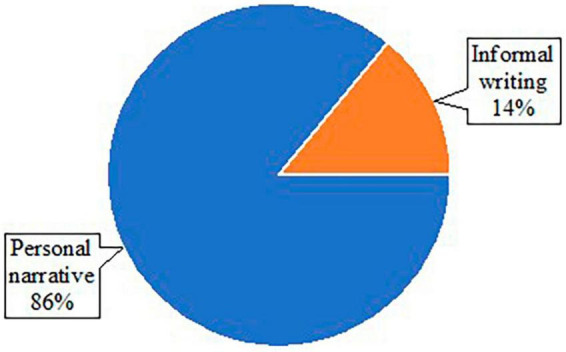
Pie chart of types of stimuli within studies without use of pictures.

**FIGURE 4:**
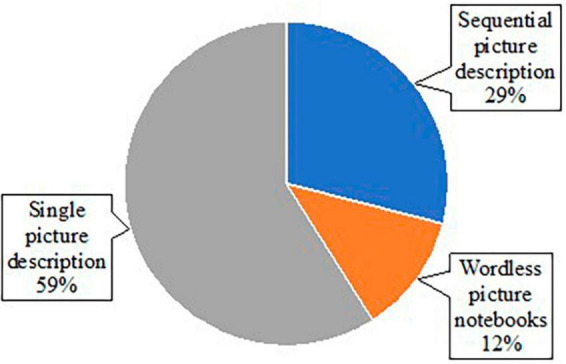
Pie chart of types of pictures used within studies using picture stimuli.

All studies reviewed used some task to elicit written discourse samples for analysis (Table 1). Broadly, discourse tasks can be categorized into two formats: either with or without the presentation of picture stimuli. In some cases, both formats were used to elicit different types of written discourse samples. Fourteen studies included picture stimuli (e.g., the Cookie Theft Picture), while seven studies elicited writing samples without pictures (e.g., asking participants to write about a memorable moment) (Figure 2). For the studies eliciting writing samples, only 1 study used a format of letter writing (e.g., writing a letter to friends), and six studies used personal narratives (Figure 3). Of the 14 studies using picture stimuli, 10 studies included a single picture description task, and five studies used sequential pictures of two to four frame cartoons. Two studies used a wordless picture book (Figure 4). Of note, some studies used single and sequential pictures. The number of studies utilizing discourse tasks sorted by research question can be found in Table 2.

**TABLE 1:** Studies organized by participant group with corresponding discourse elicitation stimuli and discourse writing modality.

References	Discourse elicitation task	Discourse writing modality
**Studies that included people with aphasia**			
Behrns et al., 2010	One personal narrative, “I have never been so afraid” Wordless picture book “Frog where are you?”	Typed
Behrns et al., 2008	Personal narrative, “I have never been so afraid”	Typed
Behrns et al., 2009	Personal narrative, “I have never been so afraid”	Typed
Johansson-Malmeling et al., 2020	Wordless picture book “Frog where are you?” Personal narrative, “Last time I made someone happy”	Typed
Vandenborre et al., 2018	Picture description, stimuli included the line drawing from the Dutch version of the comprehensive aphasia test	Handwritten
**Studies that included people with Alzheimer’s disease**			
Pekkala et al., 2013	Description of the cookie theft picture	Handwritten
Rodríguez-Ferreiro et al., 2014	Description of a three picture sequence (man preparing to fish)	Handwritten
Forbes-McKay et al., 2014	4 picture description tasks total—2 simple pictures (cookie theft and tripping woman), 2 complex pictures (traffic chaos and bus stop)	Handwritten
Forbes-McKay and Venneri, 2005	4 picture description tasks total—2 simple pictures (cookie theft and tripping woman), 2 complex pictures (traffic chaos and bus stop)	Handwritten
Forbes et al., 2004	4 picture description tasks total—2 simple pictures (cookie theft and tripping woman), 2 complex pictures (traffic chaos and bus stop)	Handwritten
Groves-Wright et al., 2004	Description of the cookie theft picture	Handwritten
**Studies that included people with mild cognitive impairment**			
Aramaki et al., 2016	Personal narrative, participants were asked to write and speak about one of the happiest events of their lives	Handwritten
Smolík et al., 2016	Personal narrative, participants were asked to write 12–15 sentences about a recent and past event	Handwritten
Kim et al., 2022b	Cookie theft picture	Handwritten
**Studies that included multiple patient populations**			
Mortensen, 2005	Writing an informal letter	Handwritten
Davy et al., 2016	Description of the picnic	Handwritten
Hayashi et al., 2015	4-frame cartoon (supplementary test from the standard language test of Aphasia)	Handwritten
Sitek et al., 2015	Cookie theft picture, river scene, beach scene	Handwritten
Sand Aronsson et al., 2020	Cookie theft picture	Handwritten

**TABLE 2:** The number of studies utilizing discourse tasks sorted by research question.

Tasks	Number of studies			
	RQ1	RQ2	RQ3	RQ4
Single picture description	6	4	3	3
Sequential picture description	4	2	1	1
Wordless picture books	2			
Personal narrative	6			3
Informal letter writing	1	1		

### 3.4 Measures used across studies

In the 19 articles reviewed, there are 102 different measures used to quantify or describe various writing behaviors. In Table 3, measures were grouped into nine categories due to a large number of measures. The most frequently utilized measure was the total number of words (*n* = 7), followed by number of different words, phrase length, grammatical form, pictorial theme, spelling errors, error monitoring, phonemic paragraphias, semantic paragraphias, information content, visual paragraphias, words per minute, and general quality of writing based on Goodglass et al. (2001) (*n* = 3). For the number of different words, type-token ratios (TTR) were calculated in three articles, and modified TTR were also used in two articles. However, due to the lack of description in the modified TTR in the two articles, it cannot be confirmed whether they are the same measure or not. It appears that different names were utilized to quantify the same aspect of writing performance in some of studies (i.e., target words, core words, important content words). Seventy-eight measures were used in one study, and only 24 measures were repeatedly used in two or more studies (i.e., total number of words, target words, number of important content words, number of different words, proportion of nouns, proportion of verbs, lexical density, lexical diversity, words per T-unit, phrase length, clauses per T-unit, correct sentences, grammatical form, idea density, pictorial theme, spelling errors, error monitoring, phonemic paragraphias, semantic paragraphias, information content, visual paragraphias, words per minute, two different rating scales for assessing quality of writing). It should be noted that Mortensen (2005) is a qualitative study with comprehensive description of writing performance in the study populations, and thus the measures in Table 3 were determined based on research questions mentioned in the original article. The number of studies utilizing linguistic measures sorted by research question can be found in Table 4.

**TABLE 3:** Summary of measures used in studies.

Measures	References	Description/specifications in articles
**Word-level**		
Total number of words	Behrns et al., 2008, 2009, 2010; Pekkala et al., 2013; Sitek et al., 2015; Vandenborre et al., 2018; Sand Aronsson et al., 2020	
Total number of correct words	Rodríguez-Ferreiro et al., 2014	
Target words (core words)	Pekkala et al., 2013; Kim et al., 2022b	Core words were defined as primary words which were defined as lexical items that were produced >10 times in the normative samples
Target words full	Pekkala et al., 2013	Core words plus additional words that were defined as critical lexical items that were produced between 2 and 9 times by the comparison group
Number of important content words	Hayashi et al., 2015; Vandenborre et al., 2018	Number of important content words was determined based on 7 predetermined content words with a high score of 7.
Number of different words	Rodríguez-Ferreiro et al., 2014; Aramaki et al., 2016; Davy et al., 2016^a^	Type-token ratio (TTR)
Word2Vec Distance	Davy et al., 2016	Set of words from the controls
Filtered Word2Vec clusters	Davy et al., 2016	Only words semantically related with the content of the unknown description
Brunet’s index	Davy et al., 2016	Length insensitive version of the TTR
Potential vocabulary size	Aramaki et al., 2016	Modified version of the TTR
Information units	Groves-Wright et al., 2004	New, discrete elements of content that accurately describe features of the picture.
Conciseness	Groves-Wright et al., 2004	Number of information units divided by the total number of words produced
Correct information units	Vandenborre et al., 2018	Content words and function words that are intelligible in context, accurate in relation to the picture(s) or the topic, and relevant to and informative about the content of the picture(s)
Proportion of correct information units	Vandenborre et al., 2018	
Vocabulary level	Aramaki et al., 2016	Individual’s average difficulty in lexical choices.
Number of nouns	Sitek et al., 2015	
Proportion of nouns	Sitek et al., 2015; Davy et al., 2016	
Number of verbs	Sitek et al., 2015	
Proportion of verbs	Sitek et al., 2015; Davy et al., 2016	
Proportion of adjectives	Davy et al., 2016	
Proportion of pronouns	Davy et al., 2016	
Proportion of function words	Davy et al., 2016	Number of function words over the total number of tokens
Lexical density	Behrns et al., 2009, 2010	Proportion of open-class words
Lexical diversity	Behrns et al., 2009, 2010	Computer program Vocab used
Words per T-unit	Behrns et al., 2009, 2010	Wolfe-Quintero et al., 1998
Useful strokes	Behrns et al., 2008	Total keystrokes that were left in the edited text
**Grammatical/syntactic level**		
Mean length of utterances	Johansson-Malmeling et al., 2020	Calculated by dividing the total number of words by the number of utterances.
Phrase length	Forbes et al., 2004; Forbes-McKay and Venneri, 2005; Forbes-McKay et al., 2014	To measure the longest run of uninterrupted speech
Number of sentences	Sitek et al., 2015	No specific descriptions provided
Complex sentences	Sitek et al., 2015	No specific descriptions provided
Maximum sentence length	Sitek et al., 2015	No specific descriptions provided
Min-max sentence length	Davy et al., 2016	Average of the length of all sentences occurring in the description.
Clauses per T-unit	Behrns et al., 2009, 2010	Ulatowska et al., 1978
Number of T-unit	Rodríguez-Ferreiro et al., 2014	No specific descriptions provided
Correct sentences	Sitek et al., 2015; Vandenborre et al., 2018	Sentence with all the arguments required by the verb correctly inserted
Compound sentences	Vandenborre et al., 2018	Sentence that contains two or more independent clauses joined by a coordinating and/or subordinating conjunction and sentences which contain one or more relative clauses
Grammatical index	Vandenborre et al., 2018	Calculated by dividing the number of correct compound sentences by the number of utterances and then multiplying this value by 100
Grammatical form	Forbes et al., 2004; Forbes-McKay and Venneri, 2005; Forbes-McKay et al., 2014	Presence of an appropriate use of syntactic conjunctions, tenses, conditionals, subordinate clauses and passive constructions
Syntactic complexity	Sand Aronsson et al., 2020	Average dependency distance
Idea density	Sand Aronsson et al., 2020	Number of propositions in a given text
	Davy et al., 2016	Total number of assertions or claims whether true or false, in a proposition.
Propositional density	Smolík et al., 2016	All main verbs, adjectives, adverbs, prepositions, numerals, and connectives.
Dependency distance	Aramaki et al., 2016	Metric that demonstrates the average dependency distance for each phrase in a narrative.
Yngve score	Aramaki et al., 2016	Number of branches for each node represents the number of arguments for a phrase.
**Discourse-level**		
Cohesive markers	Vandenborre et al., 2018	Word that connects the meaning expressed in the ongoing utterance with that already expressed, or which will be expressed in subsequent utterances
Proportion of cohesive markers	Vandenborre et al., 2018	
Proportion of cohesive adequacy	Vandenborre et al., 2018	Calculated as a percentage by dividing the number of correct ties by the total number of ties and multiplying this value by 100.
Coherence	Behrns et al., 2010	Protocols from Coggins et al. (1998) used.
Text structure	Behrns et al., 2010	Protocols from Coggins et al. (1998) used.
Number of main concepts	Groves-Wright et al., 2004	Counting the number of main concepts in the picture described by the subject.
Pictorial theme	Forbes et al., 2004; Forbes-McKay and Venneri, 2005; Forbes-McKay et al., 2014	Number of observable actions identified by each participant
Ideational metafunction	Mortensen, 2005	Language content (e.g., number of topics)
Interpersonal meanings	Mortensen, 2005	Interaction between writer and reader
Narrative efficiency	Groves-Wright et al., 2004	Overall scores based on three efficiency measures (adequate description and sequencing of events, narrative conciseness, and relevance of information)
**Error-related**		
Word level errors	Behrns et al., 2009	Any errors including misspellings and semantic substitutions
Lexical errors	Hayashi et al., 2015	Include substitution, omission, letter displacement, incorrect sonant marks, and other types of mistakes
Spelling errors	Behrns et al., 2010; Davy et al., 2016; Johansson-Malmeling et al., 2020	The number of words in the final text containing one or more spelling errors divided by the total number of words in the final text (expressed as a percentage) in the narratives.
Error monitoring	Forbes et al., 2004; Forbes-McKay and Venneri, 2005; Forbes-McKay et al., 2014	Proportion of errors that are detected and corrected
Phonemic paragraphias	Forbes et al., 2004; Forbes-McKay and Venneri, 2005; Forbes-McKay et al., 2014	Proportion of phrases containing phonemically erroneous substitutions
Letter errors	Sitek et al., 2015	Sum of spelling errors (deletions, insertions, substitutions, transpositions)
Deletions	Sitek et al., 2015	No specific descriptions provided
Insertions	Sitek et al., 2015	No specific descriptions provided
Substitutions	Sitek et al., 2015	No specific descriptions provided
Transpositions	Sitek et al., 2015	No specific descriptions provided
Effect of part of speech	Behrns et al., 2010	Total number of incorrectly written content words divided by the total number of content words
Effect of position in word	Behrns et al., 2010	Proportion of word-level errors in the word stem and the affix
Semantic substitutions	Behrns et al., 2010	Correctly spelled words that does not have the correct meaning (different from the target word)
Semantic paragraphias	Forbes et al., 2004; Forbes-McKay and Venneri, 2005; Forbes-McKay et al., 2014	Proportion of phrases containing semantically erroneous substitutions
Substitution of function words	Behrns et al., 2010	Correctly spelled function words that is incorrect in context
Morphological errors	Behrns et al., 2010	An example indicated errors in suffix
Neologisms	Behrns et al., 2010	Word meaning is not possible to trace
Language errors	Vandenborre et al., 2018	Including phonemic errors, verbal phonological errors, verbal semantic errors, unrelated errors, morphological errors and neologisms
Proportion of language errors	Vandenborre et al., 2018	
Information content	Forbes et al., 2004; Forbes-McKay and Venneri, 2005; Forbes-McKay et al., 2014	Proportion of phrases containing, indefinite terms, inappropriate phrases and redundant words
Syntactic errors	Hayashi et al., 2015	Include incomprehensible sentences missing a subject or a verb, incorrect verb argument, verb inflections
Visual paraphasias	Forbes et al., 2004; Forbes-McKay and Venneri, 2005; Forbes-McKay et al., 2014	Proportion of phrases containing, word substitutions that are visually similar to the target object
**Writing speed**		
Total time	Pekkala et al., 2013	
Words per minute	Behrns et al., 2008; Johansson-Malmeling et al., 2020	
Time to write target words	Pekkala et al., 2013	Time divided by total target words
Time sent to edit	Behrns et al., 2008	Mean time for the subject to make the edit, successful or failed
Time for trial and error	Behrns et al., 2008	Mean time for the subject to make the edit failed.
Active typing time	Behrns et al., 2008	Typing time excluding pause time
Typing speed	Johansson-Malmeling et al., 2020	Number of words divided by time on tasks
**Editing ability**		
Edited part of speech	Behrns et al., 2008	Whether the edited word is content word or function word
Edited position in word	Behrns et al., 2008	Editing occurred within the word stem or affix
Edited position in sentence	Behrns et al., 2008	What number of the word was edited in the sentence, e.g., if there was a sentence with six words, the initial word = 0%; the third word = 40%; the last word = 100%.
Word level edits	Behrns et al., 2008	Proportion of words that had been edited
Successful edits	Behrns et al., 2008	Proportions of all words that were edited to form a correct word
Failed edits	Behrns et al., 2008	Incorrectly written word that was still incorrect after editing
Proportion of un-edited text	Johansson-Malmeling et al., 2020	The total number of “tokens” in the final text divided by the total number of instances of the pressing of token keys
Instant edit after letter	Behrns et al., 2008	Proportion of all edits that were instant and edited right after the incorrect letter.
Long-distance edit	Behrns et al., 2008	Proportion of all edits that were long-distant.
Edit strategy trial and error	Behrns et al., 2008	Proportion of all edits that were edited in a trial and error way.
**General quality of writing**		
Rating scale	Behrns et al., 2009	Protocols from Olness et al. (2005): read written stories and evaluate the quality of them using questionnaire
Rating scale	Hayashi et al., 2015; Vandenborre et al., 2018	A general score was given on a scale from 1 to 6 (1 representing severe deficiency and 6 representing normal performance) based on the number of content words, grammatical errors, and character errors.
Rating scale	Forbes et al., 2004; Forbes-McKay and Venneri, 2005; Forbes-McKay et al., 2014	Modified 7-point likert scale based on Goodglass et al. (2001)
**Formatting**		
Frame	Mortensen, 2005	Addressing the recipient
Omission of diacritical marks	Sitek et al., 2015	No specific descriptions provided
Punctuation errors	Sitek et al., 2015	No specific descriptions provided
General structure	Mortensen, 2005	Impression of the completeness; integrity ad coherence of the overall formatting in response to the purpose of writing. Subcategories—date; salutation; frame; News; L-taking; Sign-off.
Engagement (semantic organization)	Mortensen, 2005	Interaction function (Writer’s interpersonal and ideational orientation); Engagement in frame (Writer’s orientation to the purpose and content of the letter); Engagement as leave-taking (closing move)
**Others**		
NGrams	Davy et al., 2016	Lexical regularities hidden in the writing style of an author and its syntactic complexity (computed in natural language processing)

T-unit consists of a main clause plus any clauses subordinate to it.

^a^In the original article, authors used a term, vocabulary size.

**TABLE 4:** The number of studies utilizing linguistic measures sorted by research question.

Measures	Number of studies			
	RQ1	RQ2	RQ3	RQ4
Word-level	11	4	2	4
Grammatical/syntactic level	10	3	1	5
Discourse level	5	3	1	3
Error-related	9	3	1	3
Writing speed	3		1	
Editing ability	2			
General quality of writing	6	2	1	3
Formatting	1	2		

### 3.5 Summary of results

This review identified 19 studies that focused on discourse-level writing performance in PWA, PPA, MCI, and AD. The summary section has been organized by research question. Findings in studies reviewed are summarized in Table 5.

RQ1. What are the documented differences in written discourse when comparing PWA, PPA, MCI, and AD to control groups?

**TABLE 5:** Summary of literature.

References	Population (*N*, mean age)	Age^a^	Edu^b^	Results and interpretation
Behrns et al., 2010	PWA (8;42.5) Control group (10, 23.5)	**✗**	n.m	$PWA produced fewer **total words** (*p* = 0.013), had more **word level errors** (*p* = 0.05), produced fewer **words per T-unit** (*p* = 0.041), and fewer **clauses per T-unit** (*p* = 0.003). Average **text structure scores** were significantly lower for PWA (*p* = 0.001) and **coherence scores** were significantly lower (*p* = 0.011). $For both groups, more **errors** were found in the word stem than the affix. No significant difference in **lexical density** (*p* = 0.242), **lexical diversity** was significantly lower for PWA in the “frog story” (*p* = 0.0003)
Behrns et al., 2008	PWA (8, 42.5) Control group (10, 23.5)	**✗**	n.m	$No significant difference in **useful strokes**, however, there was a significant difference in proportion of **edited words** and the proportion of **successfully edited words.** PWA used a significantly higher proportion of **trial-and-error edits** than the control group. $PWA required more **time to edit** (21.5 s) compared to the control group (5.3 s). **Failed edits** for PWA (43.5 s) were more time consuming than successful edits. $A factor that influenced the success of edits was the **errors position in the word**. Errors in the affix were less likely to be corrected (*p* < 0.05).
Behrns et al., 2009	PWA (8, 42.5) Control group (10, 23.5)	**✗**	n.m	$Controls produced significantly more **words per t-unit** than PWA group (*p* = 0.006). $Controls had significantly more **clauses per t-unit** than PWA group (*p* = 0.025) and there was a significant interaction effect (*p* = 0.004) @PWA had significantly more **word-level written errors** than Controls group (*p* = 0.028) **Clauses per t-unit** were significantly higher in written than spoken for Controls group but not for PWA group @PWA group written narratives rated significantly more **coherent and easier to understand** than spoken narratives
Johansson-Malmeling et al., 2020	PWA (16, 69.13) Control group (26, 67.96)	✓	✓	**$Production rate** was significantly slower for PWA in picture-elicited (*p* < 0.001) and free narrative (*p* < 0.001) $**Proportion of unedited text** was significantly lower for PWA in picture-elicited (*p* = 0.004) and free narrative (*p* = 0.004) $PWA produced significantly **shorter texts** in both tasks (*p* < 0.001) and significantly more **spelling errors** in the picture-elicited task (*p* = 0.01)
Vandenborre et al., 2018	PWA (50, 63.4) Control group, people without stroke (50, 63.8)	✓	✓	$PWA produced fewer **total words** (*p* = 0.00), and had shorter **MLU** (*p* = 0.00) $PWA produced fewer **CIU**s (*p* = 0.00) and lower **proportion of CIUs** (*p* = 0.00) $PWA produced fewer **correct written sentences** (*p* = 0.00), fewer **compound sentences** (*p* = 0.00) and lower **grammatical index** in writing (*p* = 0.00) $PWA made more **errors** (*p* = 0.00) and had a higher **proportion of language errors** (*p* = 0.00) $PWA had less **cohesive adequacy** (*p* = 0.00) and **fewer cohesive markers** (*p* = 0.00) but **proportion of cohesive markers** was not significantly different (*p* = 0.99) @**Total number of words** significantly lower for PWA, and associated with **severity**, in written but not in spoken modality @**Percentage of language errors** significantly higher in written than spoken modality for PWA, vice versa for healthy controls @**Cohesive markers** differentiated PWA and controls in written but not in spoken modality
Mortensen, 2005	PWA (10, 56.3) TBI (15, 26.8) Control group, people without brain damage (15, 41.4)	✓	✓	$No statistical findings were reported $**Frame** was selected by 50% of writers with aphasia and 80% of the control group $PWA were less **complex** in their writing and the control group was the most consistently complex $**Leave taking** was used by 60% of PWA and 93% of controls $PWA produced fewer **topics** than the control group $PWA had reduced content and ideational resources #Greater **semantic representation** by including diverse comments and closures in TBI than in PWA #Less use of **frame and leave-taking** in letter in PWA than in TBI #Less **language content** in letter in PWA than in TBI
Pekkala et al., 2013	AD (23, 72) Controls (24, 70) multiple assessment (Time 1,2,3)	✓	✓	$Significant differences in **total number of words** (*p* = 0.026), **total number of target words** (*p* = 0.01), **time divided by total target words** (*p* = 0.028), **target words full** (target words plus additional words) (*p* < 0.001) ?Greater difference in **target words full** was found between the two groups at Time 1 and Tie 2 ?No statistical report on the AD group over time
Rodríguez-Ferreiro et al., 2014	Probable AD (22, 75.4) Control group (22, 75.4)	✓	✓	$People with AD produced significantly fewer **T-units** (*p* < 0.005).
Forbes-McKay et al., 2014	Min-mod AD (31, 76.03) People with AD who were re-evaluated (15, baseline-6–12 months) Controls (30, 78.25)	✓	✓	$Significantly different **phrase length, grammatical form**, and **information content** for simple and complex pictures $People with AD produced more **phonemic paragraphias** and **semantic errors** than the control group in complex but not simple pictures. $There was also a significant difference in **error monitoring** and **pictorial themes** included for the complex in simple pictures (*p* < 0.005) ?**Visual paragraphias** at 12 months for simple tasks in AD (*p* = 0.001; η^2^ = 0.39)
Davy et al., 2016	AD or MCI (47, NA) Controls (154, NA)	n.m	n.m	**$Lexical and semantic irregularities** were able to discriminate healthy adults from cognitively impaired patients at 86.1% accuracy.
Forbes-McKay and Venneri, 2005	Probable AD (30, 73.98) Controls (240, 51.61)	**✗**	**✗**	$The highest proportion of people with AD to score below the cut-off were in the complex picture description task in the areas of **pictorial themes** (67%), **information content** (87%), **error monitoring** (46%) and phonemic paragraphias (43%). $In the simple picture description task the highest proportion of people with AD to score below the cut-off were in the areas of **pictorial themes** (50%), **information content** (40%), **error monitoring** (36%), and **semantic paraphasias** (27%) @Classification of patients vs. controls numerically better based on spoken compared to **written outcomes**
Forbes et al., 2004	Probable AD (30; 75.83) Minimal group (10; 74.1) Mild group (10, 78.5) Moderate group (10, 74.9) Control group (30, 78.3)	n.m	n.m	$Significantly **shorter phrase length** (*p* < 0.001) and **less complex grammatical form** (*p* < 0.001). People with AD produced significantly more **semantic** (*p* < 0.05) and **phonemic paragraphias** (*p* < 0.01) $People with AD produced less **information content** (*p* < 0.05) and fewer **pictorial themes** (*p* < 0.05) $Significant main effect for the **mechanics of writing** scale (*p* < 0.001) and words containing **stroke errors** (*p* < 0.05) #The moderate AD group had more **letter formation difficulties** than the minimal and mild AD groups (*p* < 0.05) #**Writing style** changes from cursive to print between the minimal AD and moderate AD groups (*p* < 0.05)
Groves-Wright et al., 2004	Mild AD (14, NA) Moderate AD (14, NA) Control group (14, NA)	✓	✓	$Significant main effect for group (*p* < 0.001) for the **global picture description score**. #The group with moderate AD scored significantly lower than the group with mild AD in the written picture description task (*p* < 0.001) (**global picture description**) @Written picture description more impaired than spoken for moderate (*p* < 0.001) but not mild AD or healthy controls (**global picture description**)
Hayashi et al., 2015	aMCI (25, 76); mild AD (38, 77.4) Control group (22, 74.7)	✓	✓	$Both AD (*p* = 0.001) and aMCI (*p* = 0.032) group scored lower than controls on the **total writing score** $AD group had significantly lower **information content** than the control group (*p* = 0.041). #Significant difference in **Kana errors** between the control and AD group (*p* = 0.012) #Higher **lexical error** scores in AD than in MCI (*p* = 0.011)
Aramaki et al., 2016	MCI (7, 80.25) Controls (14, 77.21)	**✗**	**✗**	$N.S for **TTR, PVS, VL, DepD, Yngve** @Written **narratives** longer than spoken for MCI but not for controls @Gap in content between written and spoken narratives revealed a larger **vocabulary** for MCI
Smolík et al., 2016	aMCI (20, 72.05) Controls (20, 72.05)	✓	✓	$No differences in propositional density @Only spoken narratives of childhood memories differentiated between MCI and controls (null results for written narrative measures)
Kim et al., 2022b	Stable MCI (45, 69.3); Converters (26, 70.3) ⋅ multiple assessment (baseline-Time1)	✓	✓	?**Core words** (Stable MCI > Converters) (*p* = 0.004)
Sitek et al., 2015	LPA (9, 70); AD (13, 77); MCI (13, 67)	**✗**	✓	#**Number of verbs** (LPA > AD) (*p* = 0.008) #**Number of insertion errors** (LPA > AD; LPA > MCI) (*p* = 0.02) #**Number of sentences** (LPA > AD) (*p* = 0.025)
Sand Aronsson et al., 2020	SCI (28, 58.7); MCI (41, 62.5); AD (45, 67.4)	**✗**	**✗**	#**Syntactic complexity** was a significant predictor of cognitive diagnosis (average dependency distance) (*p* < 0.001)

PWA, people with aphasia; SCI, subjective cognitive impairment; MCI, mild cognitive impairment; aMCI, amnestic MCI, stable MCI, those initially diagnosed with MCI were classified with MCI at a subsequent time point; Converters, those initially diagnosed with MCI progressed to dementia at a subsequent time point; AD, Alzheimer’s disease; LPA, Logopenic primary progressive aphasia; TBI, traumatic brain injury; n.m, not mentioned. Results and conclusions related to each research questions are denoted by the following: RQ 1 = $ RQ 2 = #; RQ 3 = ?; RQ 4 = @.

^a^Indicates whether the study used age-matched groups.

^b^Indicates whether the study used education-matched groups.

*RQ 1-1. Aphasia vs Controls.* Across the articles on written discourse performance in PWA, there was variability in the discourse elicitation tasks selected, written discourse measures, and use of the handwritten and typed modalities. Some of the tasks used to elicit written discourse included wordless picture book prompts, personal stories (e.g., “I have never been so afraid”), a personal letter, and picture descriptions. Discourse measures also varied with five of the six papers evaluating at least one aspect of written discourse microstructure (Behrns et al., 2008, 2009, 2010; Vandenborre et al., 2018; Johansson-Malmeling et al., 2020) and four of the six papers including at least one measure of discourse macrostructure (Mortensen, 2005; Behrns et al., 2009, 2010; Vandenborre et al., 2018).

Although the methods used to evaluate written discourse varied across studies, there were some consistent findings that separated the performance of PWA from a control group and that provide insight into the written discourse of PWA. For example, PWA typically produced fewer words in their written discourse when compared to a control group (Behrns et al., 2010; Vandenborre et al., 2018; Johansson-Malmeling et al., 2020), and produced shorter and syntactically simpler utterances (Behrns et al., 2009, 2010; Vandenborre et al., 2018). Two papers examined the editing process or the efficiency of handwriting/typing in PWA. Results consistently suggested that PWA make more word-level errors and require more time to edit (Behrns et al., 2008; Johansson-Malmeling et al., 2020). PWA were also found to use trial-and-error approaches to editing more frequently than a control group (Behrns et al., 2008) and were less efficient in their ability to convey informative content (Vandenborre et al., 2018). While the majority of papers focused on microstructure, some also evaluated components of macrostructure such as cohesion, letter structure and story ratings from unfamiliar listeners. Cohesive adequacy was rated significantly lower for PWA (Behrns et al., 2009) and they used fewer cohesive markers; however, Vandenborre et al. (2018) found that the proportion of cohesive markers was not significantly different from controls. Mortensen (2005) evaluated written discourse performance at the word level, and found that PWA included the majority of obligatory letter elements, but the writing of PWA was less complex and included fewer topics. PWA were rated significantly lower on a number of other variables such as having adequate choice of words, enjoying telling a story and ease of understanding; however, these ratings included both spoken and written narrative comparisons with the control group (Behrns et al., 2009).

There were also some variables that were similar in the written discourse of PWA and control groups. Lexical density was not different across groups (Behrns et al., 2009, 2010) and there were mostly non-significant differences when comparing lexical diversity.

*RQ 1-2. MCI vs. Controls.* Despite the limited number of studies, there is a clear pattern in the review that no significant differences were found between MCI and control groups. Both studies utilized similar writing tasks in which the study participants described events that occurred in their lives. Aramaki et al. (2016) study, written discourse samples were obtained, asking participants to describe a happy event. In Smolík et al. (2016) study, descriptions were related to a recent memory, and a past memory from childhood. None of these studies found significant differences, but this reflects the clinical importance of the choice of written discourse elicitation tasks. Although this was a frequently used written discourse task for people with MCI, written discourse samples elicited from recounts may not be sensitive enough to capture subtle linguistic changes. Possibly, recounts that are associated with individuals’ personal life result in great variability in language samples across individuals (Wright and Capilouto, 2012). Because this task allows more latitude by its nature, writers with subtle cognitive impairment can employ compensatory strategies for accommodating their language difficulties, such as using synonyms or providing limited details. Indeed, there is some overlap in the quality of written discourse performance of MCI and control groups. In future studies, written discourse should be evaluated in multiple discourse tasks.

*RQ 1-3. AD vs Controls.* Eight studies evaluated the performance of people with AD in comparison to a control group. These studies evaluated written discourse using the handwritten modality and all implemented some method of picture elicitation task, although there was variability in the number of pictures and the complexity (i.e., describing a picture sequence vs. a single picture). The discourse measures varied, but many patterns were observed across the study findings.

The most consistent findings were that the written discourse of people with AD contained less information content (Forbes et al., 2004; Groves-Wright et al., 2004; Forbes-McKay and Venneri, 2005; Pekkala et al., 2013; Hayashi et al., 2015) and fewer pictorial themes (Forbes et al., 2004; Forbes-McKay and Venneri, 2005; Forbes-McKay et al., 2014) than the written discourse of the control group. Another apparent pattern in the written discourse of people with AD was the higher frequency of lexical and semantic errors (Forbes et al., 2004; Forbes-McKay and Venneri, 2005; Forbes-McKay et al., 2014; Hayashi et al., 2015; Davy et al., 2016). Phonemic paragraphias were also noted as sensitive for distinguishing between the written discourse of people with AD and a control group (Forbes-McKay and Venneri, 2005; Forbes-McKay et al., 2014). Two studies found that people with AD were less able to monitor for errors (Forbes-McKay and Venneri, 2005; Forbes-McKay et al., 2014). Additionally, two studies evaluated productivity of words or utterances and found that people with AD produced significantly less written discourse than the control group (Pekkala et al., 2013; Rodríguez-Ferreiro et al., 2014), and two studies reported that people with AD have reduced utterance length or complexity (Forbes et al., 2004; Forbes-McKay et al., 2014).

A series of papers used simple and complex pictures to elicit written discourse from people with AD and controls. The simple pictures included seven pictorial themes and the complex scenes had eleven. Further, the complex scenes were noted to require more integration of events. These papers reported differences based on the complexity of the discourse task. People with AD had more impaired written discourse when compared to controls, with greater differences in the complex task than in the simple picture description task, suggesting an effect of cognitive and linguistic demands of the task on writing performance (Forbes et al., 2004; Forbes-McKay et al., 2014).

RQ2. Does performance on written discourse tasks/measures distinguish between different patient populations?

Studies involving different clinical populations in written discourse demonstrated unclear findings Hayashi et al. (2015) used a sequential picture task consisting of five pictures to delineate changes in written language between amnestic MCI and AD. Performance was evaluated in consideration of seven predetermined target words produced, as well as grammatical and character errors. They found that greater errors in target words and kanji characters were able to discriminate MCI from AD. Contrarily, Sitek et al. (2015) did not find any statistical differences on any of their linguistic measures between MCI and AD groups. However, Sitek et al. (2015) reported that the logopenic PPA (lvPPA) group differed from the AD group on both the number of verbs and sentences produced. For the number of spelling errors, the people with lvPPA produced insertion errors in their writing, while the MCI and AD groups did not produce any insertion errors. The authors suggested that insertion errors may help disentangle the similarities in symptoms of the three types of neurodegenerative diseases.

Three studies cross-sectionally investigated the progression of written discourse deficits from subjective cognitive impairment to AD stages (Forbes et al., 2004; Groves-Wright et al., 2004; Sand Aronsson et al., 2020). Sand Aronsson et al. (2020) reported syntactic complexity measured by an average distance between words and their syntactic dependence in phrases or sentences has a significant relationship with cognitive declines from subjective cognitive impairment to AD. Groves-Wright et al. (2004) evaluated written discourse performance in a comprehensive way, considering four different aspects (main concepts, efficiency, information units, and conciseness ratio). They found that the moderate AD group scored lower than the mild AD group. Contrarily, Forbes et al. (2004) did not find any statistical differences in linguistic aspects of written discourse (e.g., information content, syntactic measures, and use of paragraphia), while they found a change in writing style from minimal AD to mild or moderate stages. They reported that writers at the moderate stage had difficulty forming letters, and changed cursive writing to print writing compared to those at the mild stage.

A descriptive study by Mortensen (2005) had a different approach to global structure and semantic organization in people with aphasia and traumatic brain injury (TBI). The author reported a qualitative difference between the two groups. That is, less content and fewer instances of addressing the recipient and closing remarks in the group with aphasia compared to the group with TBI. It should be noted that TBI is beyond the scope of this review. There is a positive finding in relation to this research question that has implications for clinical practice, although there is no unity across studies. To some degree, it is surprising that not all of the studies reported how they calculated each of their outcome measures, and the measures utilized across the four studies varied. This makes it challenging to identify clinically useful linguistic measures in written discourse.

RQ3. Are there written discourse measures that can be used to evaluate change over time in progressive neurogenic communication disorders?

Of note is the importance of specifying variables related to the advancement of disease, such as MCI to AD. We found that semantic aspects of written discourse were one of the common outcome measures across all three studies. Pekkala et al. (2013) determined primary core words that were produced greater than 10 times in their normative samples, and then added more words that were produced between 2 and 9 times by the controls. They were categorized as Target words, and Target words full, respectively, in the article. Subsequently, Kim et al. (2022b) conducted a retrospective chart review to examine whether a converter group who were initially diagnosed with MCI but later progressed to AD demonstrated a deterioration in core word production compared to a stable group at the MCI stage. They used the same word list that Pekkala et al. (2013) developed, and revised the scoring system. For example, Kim and colleagues did not provide points for spelling errors and repetition of the same word item. Interestingly, both studies demonstrated that production of target words was reflective of degradation in cognition.

Further, Forbes-McKay et al. (2014) expanded our knowledge about the trajectory of language deficits in neurodegenerative disease. Using a variety of outcome measures, visual paragraphias (e.g., tray for plate) statistically differentiated the performance between a 12-month follow-up and the previous follow-ups in AD, and no significant difference was found in semantic paragraphia in AD over 1 year. Together, poorer semantic skills are more pervasive throughout the disease progression, and deficits in visual processing become more prominent as the disease progressed.

One factor that aids in the interpretation of these findings is that all three studies utilized the Cookie Theft picture Goodglass et al. (2001) and Forbes-McKay et al. (2014) additionally included three different line-drawing pictures. Forbes-McKay et al. (2014) intended to manipulate the level of complexity (simple vs. complex) in the picture stimuli, and the Cookie Theft picture was categorized as one of the simple tasks. Interestingly, significant differences were found between AD patients over time only with the simple picture description task. Possibly, simple picture description tasks may be appropriate for eliciting written discourse samples for longitudinal investigation from the MCI stage to a clinically more advanced pathology.

RQ4. How does written discourse performance compare to spoken discourse performance in people with acquired neurogenic disorders?

The fourth research question focused on studies that made direct comparisons between written and spoken discourse measures. These studies have both theoretical and practical implications–any evidence of differences between written and spoken outcome measures provides insight into fundamental questions about language modality, and this is true across all populations (including neurotypical control groups). Moreover, finding that written and spoken discourse measures diverge in unique ways between patients and controls might improve understanding of the nature of the underlying deficits, as well as suggest which tasks and measures may be the most sensitive for purposes of diagnosis or prognosis. For example, indications that written, but not spoken, measures differentiate between some patient group and healthy controls would seem to support the application of writing-based tasks for assessment over spoken ones. One result of our review is that too few direct comparisons (equivalent spoken and written measures assessed within the same participants) have so far been reported in the literature, but those that have (six in total reviewed here) are highly suggestive that the two modalities do provide unique insights into the deficits, especially for PWA in other words, administering both spoken and written discourse tasks seems *not* to be redundant.

Regarding the two studies of PWA that contrasted spoken and written discourse measures (Behrns et al., 2009; Vandenborre et al., 2018), they generally found that written discourse measures were as good as or better than spoken discourse measures in terms of distinguishing between PWA and healthy controls. The two studies focused on AD (Groves-Wright et al., 2004; Forbes-McKay and Venneri, 2005) were less suggestive of the written modality being diagnostic. When written measures did differentiate between patients and controls, it was typically *less* discerning than the spoken modality. Finally, the two studies that examined MCI found little to no evidence that written narratives are diagnostic (for differentiating from healthy controls) (Aramaki et al., 2016; Smolík et al., 2016). However, we note that both of those studies suggest that the written narratives, which were untimed, afforded more opportunities for the participants to plan and revise, and that this may have resulted in masking any differences compared to healthy controls. A clear implication is that written narratives should be collected in a format more similar to spoken ones, for example by ensuring that resources such as dictionaries are used equally in both modalities (if at all).

The conclusions that can be drawn are limited for several reasons, including heterogeneity of the outcome measures assessed and a lack of statistical comparisons that specifically target the question of whether written and spoken measures provide *unique* information for the purposes of diagnosis or prognosis. In particular, researchers should consider not only assessing performance in both modalities, but also analyzing their data in such a way as to provide statistical comparisons between the two (as opposed to independently analyzing each modality). For example, a number of results showed statistical differences between patient and control groups in *both* modalities–while this might seem to suggest that there is redundant information arising from the spoken and written outcomes, it does not preclude the possibility that one is more sensitive than the other to detecting differences between populations. Similarly, while there were a number of null results that suggested the written modality was less diagnostic than the spoken, no analyses were conducted to determine whether some combination of measures based on both modalities would be superior to either alone. For example, although the spoken measure-based classification model of AD patients in Forbes-McKay and Venneri (2005) outperformed the written one, it is unknown whether a classification model with both modalities together would be superior to either on its own. It is also possible that a composite measure combining the two modalities may be more useful than either alone (given that they are not perfectly correlated), and indeed one study (Aramaki et al., 2016) reported that a measure of the gap in performance between the two modalities was diagnostic of early MCI. Therefore, future work should statistically assess the two outcome measures jointly, such as by assessing unique variance explained by one modality when controlling for the other, by investigating interactions between the two modalities, or by developing aggregate scores of spoken and written performance.

## 4 Discussion

The purpose of the study was to review the existing literature related to written discourse that assists with direct and systematic replication of the existing findings and for potential clinical use. Specifically, the current review aimed to answer the following questions: (1) what are the documented differences in written discourse in PWA, PPA, MCI, and AD compared to control groups (2) does performance on written discourse tasks/measures distinguish between different patient populations (3) are there written discourse measures that can be used to evaluate changes with disease progression, and (4) how does written discourse performance compare to spoken discourse performance in acquired neurogenic disorders? Given the heterogeneity of discourse elicitation protocols and analysis methods reported across studies, our results have not been systematically synthesized. Thus, we conducted this narrative review to answer our research questions using a systematic search.

Written discourse is an emerging area with important implications for functional communication. This review has significant implications which can potentially improve our understanding of the clinical utility of written discourse analysis and provide goals for future research on written discourse. First, many of the studies reviewed reported limited methodological details. There is a wide array of outcome measures utilized across the studies, and only a few measures were consistently present in the literature. Modified TTRs from Aramaki et al. (2016) and Davy et al. (2016) may be the same measure; however, not enough information was reported to make this determination. Simultaneously, considerable variations in the terminology used to describe what was measured exist. For example, information units that Groves-Wright et al. (2004) utilized may have been the same measure as correct information units in Vandenborre et al. (2018). Currently, the lack of information is a critical barrier to replication and generalization of the findings, which is required to strengthen scientific evidence.

Second, performance at macrostructural levels has been sporadically investigated and reported. Among 102 variables identified, only 13 measures (12.7%) (discourse level and general quality of writing domains in Table 3) were related to assessing writing skills beyond the sentence level. Translating and organizing ideas into a message in writing is challenging in people with brain injury (Wheeler et al., 2014; Dinnes and Hux, 2022). As with studies regarding spoken discourse, additional efforts must be made to conduct in-depth investigations of multilevel aspects of written discourse to form a clearer picture of the mechanisms in different patient populations.

Third, there was variability in the discourse elicitation techniques across studies, which in turn affected the quality and quantity of written language samples, such as use of lexical content and discourse organization. Based on our review, there is some evidence that structured tasks (e.g., picture description tasks) may provide more opportunities to observe deficits or breakdowns in written discourse than less structured (e.g., writing personal experience) tasks in neurodegenerative disease populations. Two studies demonstrated greater production of spelling errors found in structured tasks using wordless picture books compared to when less structured tasks were given to PWA (Behrns et al., 2010; Johansson-Malmeling et al., 2020), which provides some evidence for a task effect on written discourse outcomes. However, the value of more functional discourse writing tasks that may better capture everyday writing vs. more structured tasks that may result in more diagnostic sensitivity is not clear based on the current literature. Sixteen out of 19 studies included a single type of discourse task, which does not allow us to draw conclusions about the most appropriate elicitation technique for written discourse. Thus, future work may include manipulating tasks and stimuli to directly contrast writing performances.

Further, many questions about writing format are still unanswered. The literature reviewed included studies that evaluated handwriting and typing with no direct comparisons, although the two have varied cognitive and motoric requirements. With the recent increase in mobile messaging, such as texting and instant messaging, new questions have arisen for how to evaluate written discourse and written conversation. Now that text messaging is a common communication modality and can increase communication opportunities in those with acquired neurogenic communication disorders, it may be necessary to assess texting behaviors to capture the full range and multifaceted nature of functional communication abilities in assessment. This work is only beginning to appear in the literature and will be important for the future of writing research (Kinsey et al., 2022; Lee and Cherney, 2022).

Of note is the importance of variables that influence performance in writing, such as years of education. A high level of education is thought to increase cognitive reserve which can improve outcomes after brain damage (Beausoleil et al., 2003). We found that the level of education is not a variable which contributes to writing performance in PWA (Vandenborre et al., 2018) and people with cognitive impairment (Sand Aronsson et al., 2020). Interestingly, education was found to influence writing performance in cognitively healthy adults (Forbes-McKay and Venneri, 2005; Vandenborre et al., 2018). Mortensen (2005) speculated that variation in writing performance could be attributed to different levels of education in cognitively healthy adults. However, the effect of education in writing performance in acquired neurogenic communication disorders has not been consistently studied, and evidence to support the current findings is lacking.

As mentioned by Thiel et al. (2015), standardized diagnostic tools for functional writing are lacking. Admittedly, this lack could be due to the limited methodological foundation in written discourse research. One major issue in this field is that large-scale group studies are scarce. The lack of large-scale group studies is accompanied by the tendency for writing to still be considered as a subsidiary communication channel in the clinic and in research, despite the fact that individuals with acquired neurogenic communication disorders often report written communication as a primary concern (Thiel and Conroy, 2022). In this review, we found a number of studies that included writing skills as one of the measures but downplayed them in their findings and discussion. Although infrequently done, when both written and spoken discourse tasks were administered, the findings revealed that performance in written modality was often equally as informative, and at times more informative than, the spoken modality (Behrns et al., 2009; Vandenborre et al., 2018). From a cognitive and neurobiological perspective this is unsurprising, considering that mental processes and neural substrates required to produce written text are only partially overlapping with those for spoken language production. Overall, the comparative inattention to written discourse creates a real challenge and places investigations of writing outside the mainstream of linguistic measures.

Lastly, another main weakness in the field included inconsistent testing of reliability and replication of results across studies. For example, 15 studies collected handwriting samples in this review. Eight studies reported reliabilities in their outcome measures, and only two studies reported reliabilities in word-to-word transcription. It is doubtful that handwriting from people with neurogenic disorders is clean and easy to read, and has absolute agreement among assessors. There is a need for more rigorous methodology and evaluation of writing samples for high-quality research. A summary of the key recommendations is provided in Table 6.

**TABLE 6:** Recommendations for future research.

• Spoken and written discourse should be collected in similar formats to facilitate cross-modal comparisons (e.g., access to resources such as dictionaries or time for editing should be equated across modalities). • Reliability measures should reported, which is especially for transcription of handwriting (as compared to typing). • More standardized outcome measures are needed that are applicable to *both* spoken *and* written discourse. • The increasing importance of typing/texting for real-world communication calls for increased attention to differences between printed vs. digital written discourse. • Structured tasks (e.g., picture description) may be more diagnostic than open-ended ones (e.g., recounting a life event). • Task complexity is an important factor to consider (e.g., describing pictures with multiple, interrelated events vs. fewer, unrelated events).

## 5 Limitations of the current review

We should acknowledge that the current review was limited by the small number of studies published from January 2000 to March 2023. As the focus of this study was to provide a comprehensive description of the current state of research pertaining to discourse-level writing ability in different types of acquired neurogenic communication disorders, we conducted a systematic search. However, we could not conduct a systematic review and meta-analysis due to heterogeneity in methods, study populations, tasks, and outcome measures, and thus results were summarized narratively. This study did not include any gray literature (e.g., dissertation) and included only English-written articles, which might have affected our results. Publication bias could not be estimated due to the small number of articles that targeted the acquired language disorders in this review (fewer than 10 per disorder). Some individuals with specific disorders are underrepresented in this review such as PPA, and thus, we have insufficient evidence to support clinical usability of written discourse at the current stage. Moreover, there are some articles that did not fully report demographic information on study populations, how linguistic variables were computed, how the researchers investigated reliability of the transcription, and/or calculation of linguistic variables. Therefore, data should be interpreted with caution. Lastly, nine articles (47%) are relevant to different languages (e.g., Swedish, Japanese), and the study populations were non-English speakers. In those nine articles, some measures used were originally developed in a particular language (e.g., affix in Swedish, Kana errors related to number of strokes in Japanese). The diversity of linguistic backgrounds in the articles reviewed makes it difficult to draw robust conclusions and recommendations on the feasibility of the approach and clinical practices.

## 6 Conclusion

The current review is the first attempt to provide an overview of what has been investigated in written discourse ability in people with acquired neurogenic communication disorders. It is evident from this small number studies published for each disorder that research pertaining to discourse-level writing is still at an early stage and is in need of further investigations and replications. Writing activity in daily life has a positive impact on emotions such as depression and isolation, and makes individuals feel more connected with society (Ball and Postman, 2022). Therefore, it is time to build evidence to incorporate discourse-level writing ability into assessments for our clients. We hope that this paper will encourage researchers and clinicians to apply more scrutiny to writing skills at the discourse level. Notably, this paper did not include a review of treatments that address written discourse, but this will be addressed in future work.

## Author contributions

HK: Conceptualization, Methodology, Visualization, Writing – original draft, Writing – review and editing. JO: Conceptualization, Methodology, Writing – original draft, Writing – review and editing. RW: Conceptualization, Methodology, Writing – original draft, Writing – review and editing.
