# Supplementary material for: Written discourse in diagnosis for acquired neurogenic communication disorders: current evidence and future directions
Source: Front Hum Neurosci. 2024 Jan 11;17:1264582. doi: 10.3389/fnhum.2023.1264582 (PMC10808624; doi:10.3389/fnhum.2023.1264582)
Supplement: Supplementary file 1 [file Table_1.docx]

Search Summary (Update)

| **Topic** | Aphasia and Written Discourse |
| --- | --- |
| **Reference Manager** |  |
| **Timeline** |  |
| **Restrictions or Limitations** |  |
| **Key Articles** |  |
| **Databases** | MEDLINE via PubMed, Embase, Cochrane, APAPsycInfo, Web of Science, Scopus |
| **Date Run** | February 26, 2023 |
| **Total Number of Results** | 336 |
| **Number of Duplicates Removed** | 90 |
| **Remaining Number of Results** | 246 |
| **Search Prepared By** | Marcus Spann, MLIS |

# PubMed

| Search number | Query | Results |
| --- | --- | --- |
| 10 | #9 AND (2021-2023) | 79 |
| 9 | #6 OR #7 | 823 |
| 7 | #1 OR (#2 AND #5) | 758 |
| 6 | #1 OR (#2 AND #3 AND #4) | 93 |
| 5 | "writing" [mesh] OR "narrative writing" OR "written discourse" OR "written description*" [tw] OR "descriptive writing" [tw] OR "text writing" [tw] OR "typing" | 132,265 |
| 4 | "writing" [mesh] OR "narrative writing" OR "written discourse" OR "written description*" [tw] OR "descriptive writing" [tw] OR writing [tw] OR written [tw] OR "text writing" [tw] OR "typing" [tw] | 229,110 |
| 3 | "Narration"[Mesh] OR narration [tw] OR narrative [tw] | 70,484 |
| 2 | (("Aphasia"[Mesh]) OR "Dementia"[Mesh]) OR "Cognitive Dysfunction"[Mesh] OR "aphasia" [tw] OR "Dementia*" [tw] OR "Cognitive Dysfunctions" [tw] OR "Cognitive Impairments" [tw] OR "Cognitive Impairment" [tw] OR "Mild Cognitive Impairment" [tw] OR "Mild Cognitive Impairments" [tw] OR "Mild Neurocognitive Disorder" [tw] OR "Mild Neurocognitive Disorders" [tw] OR "Cognitive Decline" [tw] OR " Cognitive Declines" [tw] | 340,994 |
| 1 | "written discourse" AND (aphasia OR dementia OR mild cognitive impairment) | 8 |

# Embase

| No. | Query | Results |
| --- | --- | --- |
| #9 | #8 AND (2021:py OR 2022:py OR 2023:py) | 31 |
| #8 | #6 OR #7 | 144 |
| #7 | #1 OR (#2 AND #3 AND #5) | 56 |
| #6 | #1 OR (#2 AND #3 AND #4) | 144 |
| #5 | 'written communication'/de OR 'writing'/exp OR 'written language'/exp OR 'narrative writing':ti,ab,kw OR 'written discourse':ti,ab,kw OR 'written description*':ti,ab,kw OR 'descriptive writing':ti,ab,kw OR 'text writing':ti,ab,kw OR 'typing':ti,ab,kw | 128330 |
| #4 | 'written communication'/de OR 'writing'/exp OR 'written language'/exp OR 'narrative writing':ti,ab,kw OR 'written discourse':ti,ab,kw OR 'written description*':ti,ab,kw OR 'descriptive writing':ti,ab,kw OR writing:ti,ab,kw OR written:ti,ab,kw OR 'text writing':ti,ab,kw OR 'typing':ti,ab,kw | 268530 |
| #3 | 'narrative'/exp OR 'narrative therapy'/exp OR narration:ti,ab,kw OR narrative:ti,ab,kw | 69073 |
| #2 | 'aphasia'/exp OR 'dementia'/exp OR 'cognitive defect'/exp OR 'aphasia':ti,ab,kw OR 'dementia*':ti,ab,kw OR 'cognitive dysfunctions':ti,ab,kw OR 'cognitive impairments':ti,ab,kw OR 'cognitive impairment':ti,ab,kw OR 'mild cognitive impairment':ti,ab,kw OR 'mild cognitive impairments':ti,ab,kw OR 'mild neurocognitive disorder':ti,ab,kw OR 'mild neurocognitive disorders':ti,ab,kw OR 'cognitive decline':ti,ab,kw OR 'cognitive declines':ti,ab,kw | 679934 |
| #1 | 'written discourse' AND ('aphasia'/exp OR aphasia OR 'dementia'/exp OR dementia OR 'mild cognitive impairment'/exp OR 'mild cognitive impairment') | 9 |

# Cochrane

Search Name: Aphasia and written discourse

Date Run: 2/26/23

Comment:

| ID | Search | Hits |
| --- | --- | --- |
| #1 | "written discourse" AND (aphasia OR dementia OR mild cognitive impairment) | 0 |
| #2 | MeSH descriptor: [Aphasia] explode all trees | 591 |
| #3 | MeSH descriptor: [Dementia] explode all trees | 7898 |
| #4 | MeSH descriptor: [Cognitive Dysfunction] explode all trees | 2809 |
| #5 | "aphasia" OR "Dementia*" OR "Cognitive Dysfunctions" OR "Cognitive Impairments" OR "Cognitive Impairment" OR "Mild Cognitive Impairment" OR "Mild Cognitive Impairments" OR "Mild Neurocognitive Disorder" OR "Mild Neurocognitive Disorders" OR "Cognitive Decline" OR " Cognitive Declines" | 38289 |
| #6 | #2 OR #3 OR #4 OR #5 | 40224 |
| #7 | MeSH descriptor: [Narration] explode all trees | 272 |
| #8 | narration OR narrative | 9029 |
| #9 | #7 OR #8 | 9029 |
| #10 | MeSH descriptor: [Writing] explode all trees | 2698 |
| #11 | "narrative writing" OR "written discourse" OR "written description*" OR "descriptive writing" OR writing OR written OR "text writing" OR "typing" | 58252 |
| #12 | #10 OR #11 | 60244 |
| #13 | MeSH descriptor: [Writing] explode all trees | 2698 |
| #14 | "narrative writing" OR "written discourse" OR "written description*" OR "descriptive writing" OR "text writing" OR "typing" | 1142 |
| #15 | #13 OR #14 | 3826 |
| #16 | #1 OR (#6 AND #9 AND #12) OR (#6 AND #15) | 310 |
| #17 | #16 AND (2021 – 2023) | 75 |

# Web of Science

TS=("written discourse" AND (aphasia OR dementia OR "mild cognitive impairment"))

OR

(TS=("aphasia" OR "Dementia*" OR "Cognitive Dysfunctions" OR "Cognitive Impairments" OR "Cognitive Impairment" OR "Mild Cognitive Impairment" OR "Mild Cognitive Impairments" OR "Mild Neurocognitive Disorder" OR "Mild Neurocognitive Disorders" OR "Cognitive Decline" OR " Cognitive Declines" ) AND TS=(Narration OR narrative) AND TS=("narrative writing" OR "written discourse" OR "written description*" OR "descriptive writing" OR writing OR written OR "text writing" OR "typing" ))

OR

(TS=("aphasia" OR "Dementia*" OR "Cognitive Dysfunctions" OR "Cognitive Impairments" OR "Cognitive Impairment" OR "Mild Cognitive Impairment" OR "Mild Cognitive Impairments" OR "Mild Neurocognitive Disorder" OR "Mild Neurocognitive Disorders" OR "Cognitive Decline" OR " Cognitive Declines" ) AND TS=("narrative writing" OR "written discourse" OR "written description*" OR "descriptive writing" OR "text writing" OR "typing"))

Timespan: All years. Indexes: SCI-EXPANDED, SSCI, A&HCI, CPCI-S, CPCI-SSH, BKCI-S, BKCI-SSH, ESCI, CCR-EXPANDED, IC.

Searched 2/26/23

54 citations

# Scopus

TITLE-ABS-KEY ( "written discourse" AND ( aphasia OR dementia OR "mild cognitive impairment" ) )

OR

( TITLE-ABS-KEY ( "aphasia" OR "Dementia*" OR "Cognitive Dysfunctions" OR "Cognitive Impairments" OR "Cognitive Impairment" OR "Mild Cognitive Impairment" OR "Mild Cognitive Impairments" OR "Mild Neurocognitive Disorder" OR "Mild Neurocognitive Disorders" OR "Cognitive Decline" OR " Cognitive Declines" ) AND TITLE-ABS-KEY ( narration OR narrative ) AND TITLE-ABS-KEY ( "narrative writing" OR "written discourse" OR "written description*" OR "descriptive writing" OR writing OR written OR "text writing" OR "typing" ) )

OR

( TITLE-ABS-KEY ( "aphasia" OR "Dementia*" OR "Cognitive Dysfunctions" OR "Cognitive Impairments" OR "Cognitive Impairment" OR "Mild Cognitive Impairment" OR "Mild Cognitive Impairments" OR "Mild Neurocognitive Disorder" OR "Mild Neurocognitive Disorders" OR "Cognitive Decline" OR " Cognitive Declines" ) AND TITLE-ABS-KEY ( "narrative writing" OR "written discourse" OR "written description*" OR "descriptive writing" OR "text writing" OR "typing" ) )

Searched 2/26/2023

62 citations

# APAPsycInfo

| **Search ID#** | **Search Terms** | **Search Options** | **Last Run Via** | **Results** |
| --- | --- | --- | --- | --- |
| S8 | S1 OR (S2 AND S3 AND S4) OR (S2 AND S5) AND (2021-2023) | Expanders - Apply equivalent subjects  Search modes - Boolean/Phrase | Interface - EBSCOhost Research Databases  Search Screen - Advanced Search  Database - APA PsycInfo | 35 |
| S7 | S1 OR (S2 AND S3 AND S4) OR (S2 AND S5) | Expanders - Apply equivalent subjects  Search modes - Boolean/Phrase | Interface - EBSCOhost Research Databases  Search Screen - Advanced Search  Database - APA PsycInfo | 526 |
| S6 | S1 OR (S2 AND S3 AND S4) | Expanders - Apply equivalent subjects  Search modes - Boolean/Phrase | Interface - EBSCOhost Research Databases  Search Screen - Advanced Search  Database - APA PsycInfo | 297 |
| S5 | DE "Written Communication" OR DE "Creative Writing" OR DE "Journal Writing" OR DE "Text Structure" OR DE "Writing Skills" OR "narrative writing" OR "written discourse" OR "written description*" OR "descriptive writing" OR "text writing" OR "typing" | Expanders - Apply equivalent subjects  Search modes - Boolean/Phrase | Interface - EBSCOhost Research Databases  Search Screen - Advanced Search  Database - APA PsycInfo | 28,185 |
| S4 | DE "Written Communication" OR DE "Creative Writing" OR DE "Journal Writing" OR DE "Text Structure" OR DE "Writing Skills" OR "narrative writing" OR "written discourse" OR "written description*" OR "descriptive writing" OR writing OR written OR "text writing" OR "typing" | Expanders - Apply equivalent subjects  Search modes - Boolean/Phrase | Interface - EBSCOhost Research Databases  Search Screen - Advanced Search  Database - APA PsycInfo | 157,388 |
| S3 | DE "Narrative Therapy" OR DE "Narratives" OR narrative OR narration | Expanders - Apply equivalent subjects  Search modes - Boolean/Phrase | Interface - EBSCOhost Research Databases  Search Screen - Advanced Search  Database - APA PsycInfo | 85,591 |
| S2 | DE "Aphasia" OR DE "Dysphasia" OR DE "Cognitive Impairment" OR DE "Dementia" OR DE "AIDS Dementia Complex" OR DE "Dementia with Lewy Bodies" OR DE "Presenile Dementia" OR DE "Pseudodementia" OR DE "Semantic Dementia" OR DE "Senile Dementia" OR DE "Vascular Dementia" OR "aphasia" OR "Dementia*" OR "Cognitive Dysfunctions" OR "Cognitive Impairments" OR "Cognitive Impairment" OR "Mild Cognitive Impairment" OR "Mild Cognitive Impairments" OR "Mild Neurocognitive Disorder" OR "Mild Neurocognitive Disorders" OR "Cognitive Decline" OR " Cognitive Declines" | Expanders - Apply equivalent subjects  Search modes - Boolean/Phrase | Interface - EBSCOhost Research Databases  Search Screen - Advanced Search  Database - APA PsycInfo | 153,471 |
| S1 | "written discourse" AND (aphasia OR dementia OR "mild cognitive impairment") | Expanders - Apply equivalent subjects  Search modes - Boolean/Phrase | Interface - EBSCOhost Research Databases  Search Screen - Advanced Search  Database - APA PsycInfo | 155 |

Search Summary

| **Topic** | Aphasia and written discourse |
| --- | --- |
| **Reference Manager** |  |
| **Timeline** |  |
| **Restrictions or Limitations** |  |
| **Key Articles** |  |
| **Databases** | MEDLINE via PubMed, Embase, Cochrane, APAPsycInfo, Web of Science, Scopus |
| **Date Run** | June 23, 2021 |
| **Total Number of Results** | 2120 |
| **Number of Duplicates Removed** |  |
| **Remaining Number of Results** |  |
| **Search Prepared By** | Stella M. Seal, MLS |

# PubMed

| Search number | Query | Results |
| --- | --- | --- |
| 9 | #6 OR #7 | 743 |
| 7 | #1 OR (#2 AND #5) | 692 |
| 6 | #1 OR (#2 AND #3 AND #4) | 72 |
| 5 | "writing" [mesh] OR "narrative writing" OR "written discourse" OR "written description*" [tw] OR "descriptive writing" [tw] OR "text writing" [tw] OR "typing" | 122,708 |
| 4 | "writing" [mesh] OR "narrative writing" OR "written discourse" OR "written description*" [tw] OR "descriptive writing" [tw] OR writing [tw] OR written [tw] OR "text writing" [tw] OR "typing" [tw] | 208,875 |
| 3 | "Narration"[Mesh] OR narration [tw] OR narrative [tw] | 52,498 |
| 2 | (("Aphasia"[Mesh]) OR "Dementia"[Mesh]) OR "Cognitive Dysfunction"[Mesh] OR "aphasia" [tw] OR "Dementia*" [tw] OR "Cognitive Dysfunctions" [tw] OR "Cognitive Impairments" [tw] OR "Cognitive Impairment" [tw] OR "Mild Cognitive Impairment" [tw] OR "Mild Cognitive Impairments" [tw] OR "Mild Neurocognitive Disorder" [tw] OR "Mild Neurocognitive Disorders" [tw] OR "Cognitive Decline" [tw] OR " Cognitive Declines" [tw] | 297,935 |
| 1 | "written discourse" AND (aphasia OR dementia OR mild cognitive impairment) | 6 |

# Embase

| No. | Query | Results |
| --- | --- | --- |
| #8 | #6 OR #7 | 124 |
| #7 | #1 OR (#2 AND #3 AND #5) | 49 |
| #6 | #1 OR (#2 AND #3 AND #4) | 124 |
| #5 | 'written communication'/de OR 'writing'/exp OR 'written language'/exp OR 'narrative writing':ti,ab,kw OR 'written discourse':ti,ab,kw OR 'written description*':ti,ab,kw OR 'descriptive writing':ti,ab,kw OR 'text writing':ti,ab,kw OR 'typing':ti,ab,kw | 118000 |
| #4 | 'written communication'/de OR 'writing'/exp OR 'written language'/exp OR 'narrative writing':ti,ab,kw OR 'written discourse':ti,ab,kw OR 'written description*':ti,ab,kw OR 'descriptive writing':ti,ab,kw OR writing:ti,ab,kw OR written:ti,ab,kw OR 'text writing':ti,ab,kw OR 'typing':ti,ab,kw | 241595 |
| #3 | 'narrative'/exp OR 'narrative therapy'/exp OR narration:ti,ab,kw OR narrative:ti,ab,kw | 51085 |
| #2 | 'aphasia'/exp OR 'dementia'/exp OR 'cognitive defect'/exp OR 'aphasia':ti,ab,kw OR 'dementia*':ti,ab,kw OR 'cognitive dysfunctions':ti,ab,kw OR 'cognitive impairments':ti,ab,kw OR 'cognitive impairment':ti,ab,kw OR 'mild cognitive impairment':ti,ab,kw OR 'mild cognitive impairments':ti,ab,kw OR 'mild neurocognitive disorder':ti,ab,kw OR 'mild neurocognitive disorders':ti,ab,kw OR 'cognitive decline':ti,ab,kw OR 'cognitive declines':ti,ab,kw | 602496 |
| #1 | 'written discourse' AND ('aphasia'/exp OR aphasia OR 'dementia'/exp OR dementia OR 'mild cognitive impairment'/exp OR 'mild cognitive impairment') | 7 |

# Cochrane

Search Name: Aphasia and written discourse

Date Run: 24/06/2021 02:03:17

Comment:

| ID | Search | Hits |
| --- | --- | --- |
| #1 | "written discourse" AND (aphasia OR dementia OR mild cognitive impairment) | 0 |
| #2 | MeSH descriptor: [Aphasia] explode all trees | 456 |
| #3 | MeSH descriptor: [Dementia] explode all trees | 6154 |
| #4 | MeSH descriptor: [Cognitive Dysfunction] explode all trees | 1754 |
| #5 | "aphasia" OR "Dementia*" OR "Cognitive Dysfunctions" OR "Cognitive Impairments" OR "Cognitive Impairment" OR "Mild Cognitive Impairment" OR "Mild Cognitive Impairments" OR "Mild Neurocognitive Disorder" OR "Mild Neurocognitive Disorders" OR "Cognitive Decline" OR " Cognitive Declines" | 32847 |
| #6 | #2 OR #3 OR #4 OR #5 | 34364 |
| #7 | MeSH descriptor: [Narration] explode all trees | 198 |
| #8 | narration OR narrative | 7964 |
| #9 | #7 OR #8 | 7964 |
| #10 | MeSH descriptor: [Writing] explode all trees | 2007 |
| #11 | "narrative writing" OR "written discourse" OR "written description*" OR "descriptive writing" OR writing OR written OR "text writing" OR "typing" | 49537 |
| #12 | #10 OR #11 | 50991 |
| #13 | MeSH descriptor: [Writing] explode all trees | 2007 |
| #14 | "narrative writing" OR "written discourse" OR "written description*" OR "descriptive writing" OR "text writing" OR "typing" | 1006 |
| #15 | #13 OR #14 | 3003 |
| #16 | #1 OR (#6 AND #9 AND #12) OR (#6 AND #15) | 262 |

# Web of Science

TS=("written discourse" AND (aphasia OR dementia OR "mild cognitive impairment"))

OR

(TS=("aphasia" OR "Dementia*" OR "Cognitive Dysfunctions" OR "Cognitive Impairments" OR "Cognitive Impairment" OR "Mild Cognitive Impairment" OR "Mild Cognitive Impairments" OR "Mild Neurocognitive Disorder" OR "Mild Neurocognitive Disorders" OR "Cognitive Decline" OR " Cognitive Declines" ) AND TS=(Narration OR narrative) AND TS=("narrative writing" OR "written discourse" OR "written description*" OR "descriptive writing" OR writing OR written OR "text writing" OR "typing" ))

OR

(TS=("aphasia" OR "Dementia*" OR "Cognitive Dysfunctions" OR "Cognitive Impairments" OR "Cognitive Impairment" OR "Mild Cognitive Impairment" OR "Mild Cognitive Impairments" OR "Mild Neurocognitive Disorder" OR "Mild Neurocognitive Disorders" OR "Cognitive Decline" OR " Cognitive Declines" ) AND TS=("narrative writing" OR "written discourse" OR "written description*" OR "descriptive writing" OR "text writing" OR "typing"))

Timespan: All years. Indexes: SCI-EXPANDED, SSCI, A&HCI, CPCI-S, CPCI-SSH, BKCI-S, BKCI-SSH, ESCI, CCR-EXPANDED, IC.

Searched 6/23/2021

218 citations

# Scopus

TITLE-ABS-KEY ( "written discourse" AND ( aphasia OR dementia OR "mild cognitive impairment" ) )

OR

( TITLE-ABS-KEY ( "aphasia" OR "Dementia*" OR "Cognitive Dysfunctions" OR "Cognitive Impairments" OR "Cognitive Impairment" OR "Mild Cognitive Impairment" OR "Mild Cognitive Impairments" OR "Mild Neurocognitive Disorder" OR "Mild Neurocognitive Disorders" OR "Cognitive Decline" OR " Cognitive Declines" ) AND TITLE-ABS-KEY ( narration OR narrative ) AND TITLE-ABS-KEY ( "narrative writing" OR "written discourse" OR "written description*" OR "descriptive writing" OR writing OR written OR "text writing" OR "typing" ) )

OR

( TITLE-ABS-KEY ( "aphasia" OR "Dementia*" OR "Cognitive Dysfunctions" OR "Cognitive Impairments" OR "Cognitive Impairment" OR "Mild Cognitive Impairment" OR "Mild Cognitive Impairments" OR "Mild Neurocognitive Disorder" OR "Mild Neurocognitive Disorders" OR "Cognitive Decline" OR " Cognitive Declines" ) AND TITLE-ABS-KEY ( "narrative writing" OR "written discourse" OR "written description*" OR "descriptive writing" OR "text writing" OR "typing" ) )

Searched 6/23/2021

288 citations

# APAPsycInfo

| **Search ID#** | **Search Terms** | **Search Options** | **Last Run Via** | **Results** |
| --- | --- | --- | --- | --- |
| S7 | S1 OR (S2 AND S3 AND S4) OR (S2 AND S5) | Expanders - Apply equivalent subjects  Search modes - Boolean/Phrase | Interface - EBSCOhost Research Databases  Search Screen - Advanced Search  Database - APA PsycInfo | 487 |
| S6 | S1 OR (S2 AND S3 AND S4) | Expanders - Apply equivalent subjects  Search modes - Boolean/Phrase | Interface - EBSCOhost Research Databases  Search Screen - Advanced Search  Database - APA PsycInfo | 274 |
| S5 | DE "Written Communication" OR DE "Creative Writing" OR DE "Journal Writing" OR DE "Text Structure" OR DE "Writing Skills" OR "narrative writing" OR "written discourse" OR "written description*" OR "descriptive writing" OR "text writing" OR "typing" | Expanders - Apply equivalent subjects  Search modes - Boolean/Phrase | Interface - EBSCOhost Research Databases  Search Screen - Advanced Search  Database - APA PsycInfo | 26,877 |
| S4 | DE "Written Communication" OR DE "Creative Writing" OR DE "Journal Writing" OR DE "Text Structure" OR DE "Writing Skills" OR "narrative writing" OR "written discourse" OR "written description*" OR "descriptive writing" OR writing OR written OR "text writing" OR "typing" | Expanders - Apply equivalent subjects  Search modes - Boolean/Phrase | Interface - EBSCOhost Research Databases  Search Screen - Advanced Search  Database - APA PsycInfo | 147,393 |
| S3 | DE "Narrative Therapy" OR DE "Narratives" OR narrative OR narration | Expanders - Apply equivalent subjects  Search modes - Boolean/Phrase | Interface - EBSCOhost Research Databases  Search Screen - Advanced Search  Database - APA PsycInfo | 75,753 |
| S2 | DE "Aphasia" OR DE "Dysphasia" OR DE "Cognitive Impairment" OR DE "Dementia" OR DE "AIDS Dementia Complex" OR DE "Dementia with Lewy Bodies" OR DE "Presenile Dementia" OR DE "Pseudodementia" OR DE "Semantic Dementia" OR DE "Senile Dementia" OR DE "Vascular Dementia" OR "aphasia" OR "Dementia*" OR "Cognitive Dysfunctions" OR "Cognitive Impairments" OR "Cognitive Impairment" OR "Mild Cognitive Impairment" OR "Mild Cognitive Impairments" OR "Mild Neurocognitive Disorder" OR "Mild Neurocognitive Disorders" OR "Cognitive Decline" OR " Cognitive Declines" | Expanders - Apply equivalent subjects  Search modes - Boolean/Phrase | Interface - EBSCOhost Research Databases  Search Screen - Advanced Search  Database - APA PsycInfo | 139,441 |
| S1 | "written discourse" AND (aphasia OR dementia OR "mild cognitive impairment") | Expanders - Apply equivalent subjects  Search modes - Boolean/Phrase | Interface - EBSCOhost Research Databases  Search Screen - Advanced Search  Database - APA PsycInfo | 147 |
